# Depression, anxiety, stress, and satisfaction with life: Moderating role of interpersonal needs among university students

**DOI:** 10.3389/fpubh.2022.958884

**Published:** 2022-09-28

**Authors:** Pei Boon Ooi, Kuan Siew Khor, Choe Chai Tan, Derek Lai Teik Ong

**Affiliations:** ^1^Department of Medical Sciences, School of Medical and Life Sciences, Sunway University, Subang Jaya, Malaysia; ^2^Future Cities Research Institute, Sunway University, Subang Jaya, Malaysia; ^3^Department of Management, Sunway University Business School, Sunway University, Subang Jaya, Malaysia; ^4^MOHE General Studies, Sunway College, Subang Jaya, Malaysia; ^5^Department of Marketing Strategy and Innovation, Sunway University Business School, Sunway University, Subang Jaya, Malaysia

**Keywords:** anxiety, depression, interpersonal needs, satisfaction with life, thwarted belongingness, stress, perceived burdensomeness

## Abstract

Depression, anxiety, and stress are ranked among the top mental health concerns faced by university students in recent times perpetuated by the proliferation of digitalization. Thus, this study was performed to assess the relationship between depression, anxiety, stress, and satisfaction with life, with interpersonal needs (perceived burdensomeness and thwarted belongingness) as moderators. A cross-sectional study using a convenient sampling method was conducted among 430 Malaysian private university students (Mean aged= 20.73 years; SD = 1.26 years). A self-administered questionnaire comprising the Depression, Anxiety and Stress Scale (DASS-21), Satisfaction with Life Scale, and Interpersonal Needs Questionnaire were used. Students who experienced lower depression and anxiety reported higher satisfaction with life under the influence of low perceived burdensomeness. Perceived burdensomeness, when coupled with depression (β = 0.76, *p* < 0.01) and anxiety (β = 0.79, *p* < 0.01), contributed 15.8% of variance in satisfaction with life. Students who experienced stress reported higher satisfaction with life under the influence of high thwarted belongingness (β = 0.73, *p* < 0.01), contributing 17.3% of the variance in satisfaction with life. For university students who experienced depression and anxiety symptoms, mental health practitioners may need to be cognizant of how to support students' education and management of their perceived burdensomeness perceptions.

## Introduction

The World Health Organization stipulates that mental health conditions among young adults are becoming a growing concern, with suicide and depression, respectively, the second and third leading cause of death among individuals aged between 15 and 29 (1). This expounds on the fact that most mental health disorders occur during young adulthood (2). Depression, anxiety, and stress are common stressors for the wellbeing of students (3), as well as their life satisfaction (4). Various studies have reported that 40 million adults in the United States have an anxiety disorder, of which 75% of them experience their first episode at age 22 which is typical during their senior college period (5). In Malaysia, the number of university students with mental health conditions has risen remarkably over the past few years with the number of people living with depression doubled and the occurrence of suicidal symptoms among students tripled over the same period (6).

The prevalence rate of mental health concerns reported by Malaysian adults has also doubled over that same period from 10.7% (1996) to 29.2% (2016) (6). While depression, anxiety, and stress were identified as being the top three mental health conditions among Malaysian students (7), this same demographic is also susceptible to developing suicidal tendencies (8) leading to poor academic performance (9). Taken together, mental health issues can significantly impact one's wellbeing and overall life performance. Thus, with almost 1.3 million Malaysian youths in college or university (10), studies on mental health conditions and the wellbeing of students are significant and crucial to promoting positive mental health among this demographic (11).

### Depression, anxiety, stress, and satisfaction with life

University students who experience higher depression, anxiety, and stress in life reported a lower level of life satisfaction (4, 12). Bukhari's study revealed that among 200 university students studied in Pakistan when surveyed, 6% suffered from depression, 5% from anxiety, and 4% from stress, and with these risk factors, the participants reported a lower level of satisfaction with life (SWL) and were more vulnerable to life challenges, such as the transition to tertiary education. On the contrary, a longitudinal 6-month study by Denovan and Macaskill (12) among 192 first-year UK participants who transited into their tertiary education showed evidence that stress at week 3 of the semester and after 6 months of the semester has a significant adverse effect on the participants' life satisfaction. Interestingly, the participants' stress levels remained relatively stable over the first year and the participants reported a higher level of unhappiness and showed a decrease in academic performance across the year. In this study, the higher education institution's (HEIs) ability and university environment to provide support and instill a sense of belongingness was quoted as potential reasons for such outcomes.

Kumar and colleagues (13) study also examined the three aspects of DASS. The results of 398 participants from three universities revealed a negative correlation between depression, anxiety, and stress with SWL. This study further revealed that 9, 34, and 13% of participants reported extremely severe levels of depression, anxiety, and stress, respectively. The psychological distress reported appeared high, especially in the anxiety domain. Students are typically young adults, and they are susceptible to positive and negative affective conditions that determine their academic performance and state of happiness or wellbeing. Hence, there is an immediate need to examine the risk factors, specifically depression, anxiety, and stress, for SWL among young adults.

Existing research has found that university students' satisfaction with life negatively correlates with depression (14). According to Seo and colleagues' study (14), 13.4% of 2,338 participants from six Korean universities had depression, according to this nationwide cross-sectional study. Life satisfaction and happiness were linked to a decreased risk of depression. In addition, it was discovered that the female with a subjective obese body image and lack of finances were strongly correlated with depressive symptoms. The results particularly imply that enhancing life satisfaction would be critical to preventing depression (14). However, this study focused on the participants' depressive symptoms, not the anxiety and stress symptoms.

A study by Guney (15) too examined the relationship between mental health status and transition of adulthood into tertiary education among 364 college Turkey students. The finding revealed that life satisfaction was negatively correlated with depression and anxiety levels. In addition, when comparing four groups (i.e., the normal, anxious, depressed, and anxious-depressed groups), the mean life satisfaction score among the normal group of participants was higher than the other three groups. Those who reported experiencing anxiety, depression symptoms, or a sense of anxious-depressed not only suffered from a lower level of life satisfaction but also a higher sense of hopelessness.

A past study also revealed that more significant anxiety is associated with greater depressive symptoms (16), where individuals exhibited greater self-criticism, hypervigilance of cues triggered by disapproval from people in their surroundings, and feelings of being unworthy of being loved. On the contrary, university students score higher in life satisfaction when their anxiety stressors are managed and reported to be low (17) due to constant monitoring, assessment, and intervention of their emotional wellbeing. Without intervention, the depression and anxiety symptoms experienced could further affect their university experience, performance, and social life. This is even more apparent with COVID-19. In the COVID-19 pandemic context, tertiary students are affected socially and academically. With this disruption, 40% of the 874 Bangladeshi students from various Bangladesh universities reported suffering from moderate-to-severe anxiety, and 72% reported depressive symptoms and anxiety living with the COVID-19 virus. These resulted in moderate to poor mental health status (18). Due to new norm disruptions brought about by COVID-19, it has undoubtedly aggravated the students' mental health status and affected their satisfaction with life.

Despite the research evidence concerning the significant effects of depression, stress, and anxiety on youths' life satisfaction, few studies focus on the relationship between depression, stress, and anxiety and life satisfaction with interpersonal needs as the moderator. However, most studies focused on the direct connection among the variables. Still, they did not consider the complexity of various factors in determining an individual's satisfaction with life. Furthermore, there are limited studies on both subdomains of interpersonal needs—that is, perceived burdensomeness and thwarted belongingness. Thus, we intend to examine the relationship and how perceived burdensomeness and thwarted belongingness, respectively, affect this relationship's strength and direction.

### The theory and role of interpersonal needs as the moderating variable

According to Maslow's Hierarchy of Needs Theory (19), individuals strive to fulfill their basic needs, such as physiological and safety needs, before striving to achieve their belongingness, esteem, and self-actualization needs. Starting from the most basic, the needs are physiological (shelter, food, and air), safety (health and university's learning environment), love and belonging needs (sense of community and friendship), and esteem (respect from peers and leadership appointment in class). To a higher level in the hierarchy is self-actualization—that is, an individual's desire to realize one's highest potential and give back to the community—for example, the ability to guide juniors or represent in high-level university events. Having most-if, not all levels of needs fulfilled or achieved leads to greater SWL (20). The first four needs are named “deficiency needs”—it is a form of deprivation needs and often time motivate individuals further when these needs are unmet (21), while the “self-actualization level” is called “growth need”—the internal motivations that drive personal growth (22). As university students have greater needs for belonging and esteem than younger students (23, 24), educators have focused on building the deficiency needs of belonging and esteem instead of the basics such as shelter or food, which are not the core responsibility of HEIs. Furthermore, failure to achieve belonging and esteem needs presented them with various life challenges and resulted in mental health issues, such as suicide ideations and suicide attempts (25, 26).

In the current study, interpersonal needs refer to individuals' desires, comprising of perceived burdensomeness and thwarted belongingness. A lacuna in the research revealed that the limitation of previous studies focuses only on psychiatric patients (27). According to past studies, perceived burdensomeness and thwarted belongingness are postulated as dynamic yet distinct measures of interpersonal needs (28, 29). These interpersonal variables fluctuate over time and are greatly influenced by interpersonal and intrapersonal factors (environment, self-beliefs, and their psychological state).

Thwarted belongingness is categorized as social or belongingness needs—an emotion when an individual feels they are not part of any social circle, such as family, friends, or another valued group (28, 30). The individuals felt disconnected from others and the absence of reciprocal care. This is the third out of the five levels illustrated in Maslow's Hierarchy of Needs (31). University students are more likely to fulfill their belongingness needs through their interpersonal relationships as they are in the developmental stages of “identity vs. role confusion” and “intimacy vs. isolation,” as suggested by Erikson (32). Their construction of self-identity and effort to achieve the feeling of belongingness, which comes from interacting with and being acknowledged by the individuals around them, contributes to this development. Failing to form close social interactions can trigger the feeling of thwarted belongingness and lead to suicidal ideation (33). To perform well academically, in relation to esteem needs, students need to first fulfill their social or belongingness needs (34). A study by Øverup and colleagues (35) presented the importance of interpersonal needs, specifically how thwarted belongingness mediates the relationship between anxiety and depressive symptoms. Their study revealed that individuals with a lower sense of belonging reported a higher level of burdensomeness, a greater sense of anxiety, and experienced greater depressive symptoms.

Perceived burdensomeness is an individual's mental state in which he or she perceives himself or herself as a burden to others (30). The perception that others would “be better off if I didn't exist” is a result of an unmet social ability connection. This mental state explains the role of individuals' innate need for connection and relatedness which allows them to grow and become competent life managers of the self (36). This unmet social ability could lead to lower SWL among young adults who may or may not have experienced symptoms of depression, anxiety, and stress (28). Thus, this present study posits that students are hindered from achieving a higher level of Maslow's Hierarchy of Needs if their belongingness needs are not fulfilled—testing the notion that thwarted belongingness functions as a moderator in this study.

Perceived burdensomeness investigated the individual self-worth in society (30), whereas thwarted belongingness is another self-belief of the psychological state where individuals desire to connect (30). These self-perceptions are the fundamental need for connectedness and belongingness—the third and fourth levels of Maslow's Hierarchy of Needs. Risk factors such as psychological distress (e.g., depression, anxiety, and prolonged stress) experienced by an individual may exert further influence on the individual's psychological wellbeing. Individuals who experienced depression, anxiety, and stress reported experiencing a lower sense of connectedness with society and a higher level of thwarted belongingness (37, 38). Under the moderation effect, thwarted belongingness and perceived burdensomeness changed the relationship's outcomes. Individuals with psychological distress, moderated with a more elevated level of thwarted belongingness, perceived burdensomeness, or a combination of both, would be prone to higher suicidal thoughts and attempts (30, 39).

**Figure 1:**
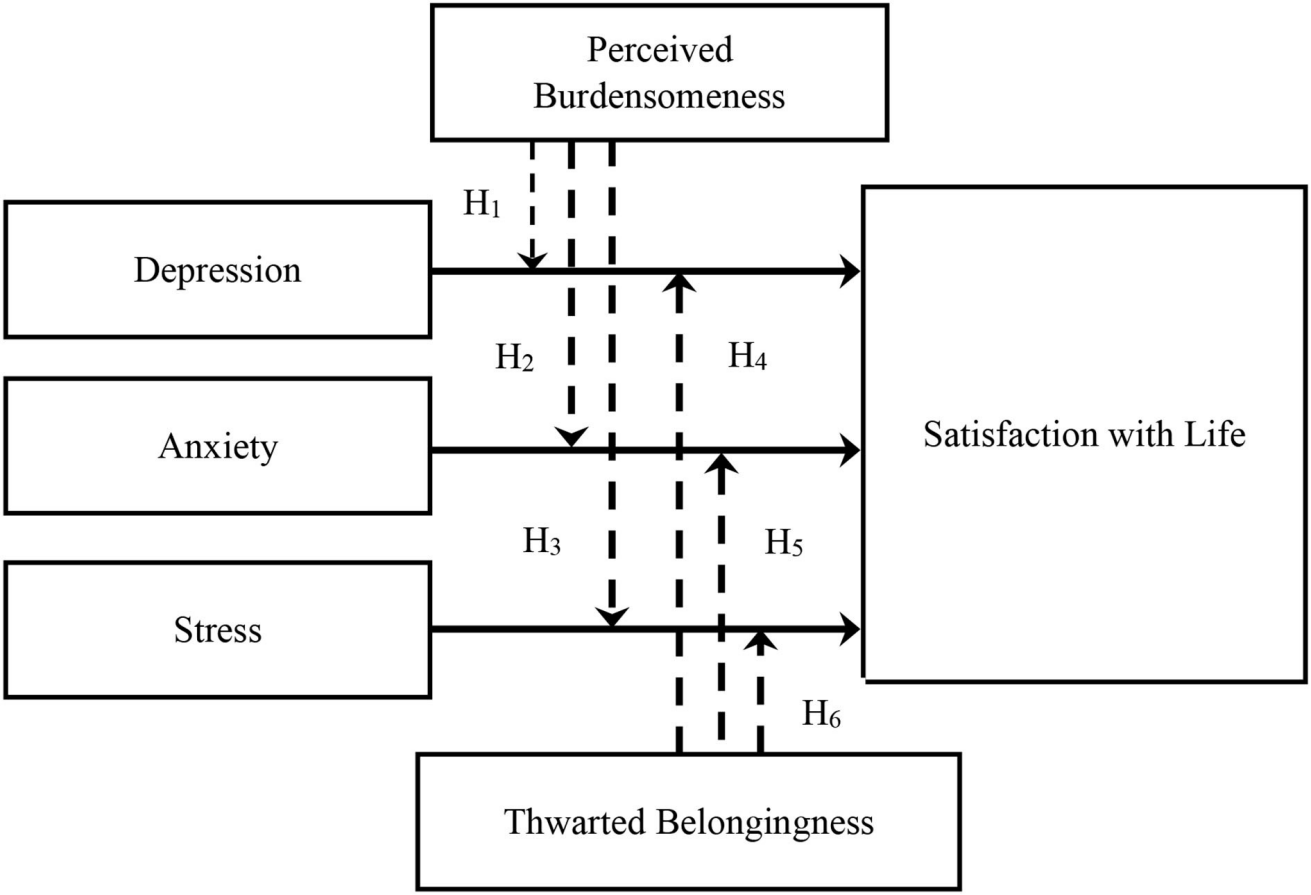
Research framework.

Depression, anxiety, and stress, paired with perceived burdensomeness and thwarted belongingness, may further affect individuals' wellbeing (40). Improving a sense of perceived burdensomeness and thwarted belongingness may improve SWL and result in lower suicidal risks (41–43). By building on the above understanding, we investigate the degrees to which thwarted belongingness and perceived burdensomeness alter the relationship between depression, anxiety, and stress and SWL. However, there are scarce studies on the moderating effect of perceived burdensomeness and thwarted belongingness on depression, anxiety, stress, and life satisfaction among Malaysian university students. Thus, this study is set to examine whether perceived burdensomeness and thwarted belongingness will moderate the relationship between depression, anxiety, stress, and SWL, before any suicidal risks, in Malaysian university students (Figure 1).

We hypothesized the following:

H_1_: Perceived burdensomeness moderates the relationship between depression and SWL.
H_2_: Perceived burdensomeness moderates the relationship between anxiety and SWL.
H_3_: Perceived burdensomeness moderates the relationship between stress and SWL.
H_4_: Thwarted belongingness moderates the relationship between depression and SWL.
H_5_: Thwarted belongingness moderates the relationship between anxiety and SWL.
H_6_: Thwarted belongingness moderates the relationship between stress and SWL.

## Methodology

### Participants

Participants (*n* = 430) were recruited from private universities in Malaysia using a convenient sampling method *via* paper self-administered questionnaires. The questionnaires in English were distributed to participants upon receiving approval from the institutional review board (IRB 2018/044). A student enumerator was hired to collect the data randomly among the private universities, and students who are aged 18 years old and above were approached to fill up the questionnaire with no compensation given. A majority of the sample (77.5%) are between the ages of 18 and 21 years old, and the breakdown of the detailed demographic characteristics is shown in Table 1.

**Table 1:** Demographic characteristics of the respondents (*n* = 430).

**Demographic variables**	**Frequency**	**%**
**Age**		
18–21 years 22–25 years	333 97	77.5 22.5
**Gender**		
Male Female	176 254	40.9 59.1
**Nationality**		
Local student Foreign student	387 42	90.0 10.0

### Data analysis

The IBM Statistical Package for the Social Sciences (SPSS) software, Version 25 was used to do the numerical analysis, bivariate correlation analysis, and regression analysis to examine the proposed hypothesis. An analysis of standard residuals was carried out on the data to identify any outliers, which resulted in four participants being removed. The post-hoc test is used to justify the moderating relationships identified for the research study.

### Measures

DASS-21 Questionnaire (DASS). DASS consists of three subscales, namely depression, anxiety, and stress. Each item in the questionnaire is rated on a four-point Likert scale, ranging from 0 (*Did not apply to me at all*) to 3 (*Applied to me very much, or most of the time*). DASS is widely used and has been validated to assess the severity of depression, anxiety, and stress among different samples (44). Depression is defined as a state of mind where the individual loses self-esteem and incentives as if believing that he or she is incapable of achieving life-defining goals (45). Anxiety is characterized as physiological hyperarousal, where the individual experiences nervousness, fearfulness, and autonomic arousal (45, 46). Stress is characterized as a negative affect or emotional state of mind, where the individual experiences persistent arousal and tension and tolerates a low threshold for frustration and becoming upset (45). DASS assesses stress as difficulty in relaxing, nervous arousal, easily upset, irritable or over-active, and impatient. One past study indicated that severe levels of depression, anxiety, and stress are highly associated with low life satisfaction among university students (4). We selected DASS-21 version as it is confirmed to exhibit good internal consistency and stable factor analysis structure to provide a desirable convergence to the study (47–50).

Interpersonal Needs Questionnaire (INQ). INQ is used to measure interpersonal needs in participants: Nine items measure thwarted belongingness, and six items measure perceived burdensomeness (28). Unlike DASS-21, items in INQ are rated on a five-point Likert scale, ranging from 1 (*Not at all true for me*) to 5 (*Very true for me*) (51). Van Orden et al. (28) suggest that thwarted belongingness and perceived burdensomeness are closely related yet highly distinctive aspects within areas of psychology (28). They also explained that INQ has been subject to multiple group analyses among younger vs. older adults and clinical vs. non-clinical samples and was found applicable to diverse populations. Previous studies mentioned that the scores derived from this scale provide good validity and psychometric properties (28). Hence, INQ is reliable enough to assess thwarted belongingness and perceived burdensomeness.

Satisfaction with Life Scale (SWLS) was developed by Diener et al. (52). It is a brief five-item instrument designed to measure the concept of life satisfaction, with each item rated on a seven-point Likert scale, ranging from 1 (*Strongly disagree*) to 7 (*Strongly agree*). In a study conducted by Swami and Chamorro-Premuzic (53) among the Malaysian population, SWLS demonstrated acceptable internal consistency reliability (Cronbach's α = 0.83).

## Data analysis and results

### Assessment of measurement items

Common method bias was examined using Harman's one-factor test to detect the existence of a single dimension that accounts for more than 50% of the variance among the measurement items (54). Table 2 presents the descriptive statistics of variables and the measurement items for DASS, INQ, and SWLS using four-point, five-point, and seven-point Likert-type response scales, respectively, to treat the effects of common method bias.

**Table 2:** Descriptive statistics of research participants.

**Score**	**Range**	**Mean (SD)**	**Skewness**	**Kurtosis**
Depression	0–3	1.89 (0.65)	0.656	0.051
Anxiety	0–3	1.83 (0.68)	0.789	0.004
Stress	0–3	2.07 (0.76)	0.343	−0.807
Perceived burdensomeness	1–5	1.74 (0.94)	1.259	0.635
Thwarted belongingness	1–5	2.86 (0.80)	−0.598	0.105
Satisfaction with life	1–7	4.06 (1.46)	−0.135	−0.722

The measurement analysis was included as common practice for social science studies. Reliability tests the consistency of instrument measures on a concept; thus, exploratory factor analysis was applied to assess the measurement items as suggested by Sekaran and colleagues (55). We assessed the measurement items to ensure that the items are reliable to the context of the study. Next, exploratory factor analysis was applied to assess the measurement items. Principal component analysis using the Varimax rotation method ensured the load of the items on the corresponding factors. The results of the factor analysis (Table 3) satisfy the Kaiser–Meyer–Olkin measure of sample adequacy (KMO-MSA) at a value above 0.6 (56), which is 0.942, and Bartlett's test of sphericity was significant at 0.000 level. Table 3 presents the final results of factor analysis, where Cronbach's alpha coefficient for all the variables was within the range of 0.847 to 0.952, which is well above the value of 0.70 recommended by Nunally (57). No items were deleted as the variables showed internal consistency. We checked whether the data met the assumption of collinearity and it indicated that multicollinearity was not a concern—the tolerance was below 1.0, and with VIF values way below the threshold of 10 (Depression, Tolerance =.45, VIF = 2.24; Anxiety, Tolerance =.45, VIF = 2.20; Stress, Tolerance =.47, VIF = 2.14; Perceived burdensomeness, Tolerance =.54, VIF = 1.85; Thwarted belongingness, Tolerance =.97, VIF = 1.03).

**Table 3:** Results of factor analysis.

**Construct**	**Item**	**Convergent validity**			**Cronbach's Alpha**
		**Loading**	**Eigenvalue**	**Variance**	
Depression	D1 D2 D3 D4 D5 D6 D7	0.605 0.667 0.711 0.680 0.722 0.549 0.571	1.485	3.62	0.890
Anxiety	A1 A2 A3 A4 A5 A6 A7	0.559 0.698 0.727 0.627 0.664 0.743 0.620	2.425	5.91	0.895
Stress	S1 S2 S3 S4 S5 S6 S7	0.766 0.700 0.760 0.764 0.727 0.760 0.650	5.578	13.60	0.924
Perceived burdensomeness	PB1 PB2 PB3 PB4 PB5 PB6	0.817 0.836 0.793 0.811 0.817 0.787	14.059	34.29	0.952
Thwarted belongingness	TB1 TB2 TB3 TB4 TB5 TB6 TB7 TB8 TB9	0.848 0.831 0.194 0.877 0.144 0.226 0.819 0.858 0.831	2.881	7.03	0.847
Satisfaction with Life	SWL1 SWL2 SWL3 SWL4 SWL5	0.859 0.893 0.893 0.891 0.828	1.570	3.83	0.938

### Hypothesis testing

Before the hierarchical regression analysis, Pearson's product-moment correlation was applied to examine the association between the variables. The strength of correlation between the variables, namely depression, anxiety, and stress, is strong and statistically significant at *r* ≥ 0.60 (Table 4). Moreover, the predictor variables appear to have stronger correlations with perceived burdensomeness than thwarted belongingness, however, reported weak but statistically significant negative correlations with SWL. We performed mean centering on the predictor variables and computed a fresh interaction term. This treatment did not change the significance of the interaction terms.

**Table 4:** Correlation between variables.

**Variables**	**ALL**					
	**(1)**	**(2)**	**(3)**	**(4)**	**(5)**	**(6)**
Depression	1					
Anxiety	0.63**	1				
Stress	0.63**	0.67**	1			
Perceived burdensomeness	0.62**	0.57**	0.54**	1		
Thwarted belongingness	0.15**	0.23**	0.24**	0.17**	1	
Satisfaction with Life	−0.28**	−0.15*	−0.15**	−0.23**	0.19**	1

^**^p < 0.01.

The moderating effects of perceived burdensomeness and thwarted belongingness were tested using a four-step hierarchical regression analysis as recommended by Sharma et al. (58, 59). Step 1 tested the effect of gender as the control variable, and it accounts for 2.3% of variance in SWL (β = −0.15, *p* < 0.01). Step 2 then tested the effects of depression, anxiety, and stress. The results showed that depression accounted for 9.9% of the variance, and the negative coefficient value indicated that depression negatively predicts SWL. Anxiety and stress were not found to be significant predictors.

Next, step 3 examined the inclusion of moderating variables (perceived burdensomeness and thwarted belongingness). Table 4 presents the regression analyses for perceived burdensomeness (left column) and thwarted belongingness (right column). The *R*-value showed no significant change with the inclusion of perceived burdensomeness to the structural path. However, with the inclusion of thwarted belongingness to the structural path, thwarted belongingness contributed R square change of 4.7% of the variance in SWL (β = 0.23, *p* < 0.01).

This study refers to Sharma et al. (58) in analyzing the moderating effects, and we proceed with Step 4 which suggests the inclusion of DAS, perceived burdensomeness, and thwarted belongingness as predictors of SWL (58). The significant interaction between depression and perceived burdensomeness (β = 0.42, *p* < 0.01) and the significant interaction between anxiety and perceived burdensomeness (β = 0.33, *p* < 0.01) contributed R change of 5.4% of variance in Step 4, both contributed 15.8% of variance in SWL. Perceived burdensomeness appears to fully moderate the relationship between depression and anxiety with SWL. Hypotheses 1 and 2 are supported, whereas hypothesis 3 is not supported.

With thwarted belongingness as the moderating variable, only stress (β = 0.56, *p* < 0.01) appeared to be a significant predictor, with an R square change of 4.7%. Hypotheses 4 and 5 are not supported, whereas hypothesis 6 is supported. Thwarted belongingness is a quasi-moderator that interacts with stress to contribute a total of 17.3% of the variance in SWL. Following these results, the post-hoc graphs are developed only for interactions that are statistically significant in the fourth step of the hierarchical regression analysis (Table 5). This step helps visualize the relationship between depression, anxiety, and stress with SWL under the moderating influence of perceived burdensomeness and thwarted belongingness.

**Table 5:** Hierarchical regression analysis: moderating effects of perceived burdensomeness and thwarted belongingness.

**Perceptions**	**Outcome**				**Perceptions**	**Outcome**			
	**Satisfaction with life**					**Satisfaction with life**			
	**Step 1**	**Step 2**	**Step 3**	**Step 4**		**Step 1**	**Step 2**	**Step 3**	**Step 4**
**Control variable**					**Control variable**				
Gender	−0.15**	−0.13**	−0.13**	−0.12**	Gender	−0.15**	−0.13**	−0.11*	−0.12**
**Predictor variable**					**Predictor variable**				
Depression		−0.32**	−0.28**	−0.57**	Depression		−0.32**	−0.31**	−0.55*
Anxiety		0.02	0.04	−0.31*	Anxiety		0.02	−0.01	0.12
Stress		0.05	0.07	0.28*	Stress		0.05	0.01	−0.54*
Perceived Burdensomeness			−0.10	−0.13	Thwarted Belongingness			0.23**	0.50**
**Interaction term**					**Interaction term**				
Depression*Perceived Burdensomeness Anxiety*Perceived Burdensomeness Stress*Perceived Burdensomeness				0.42**	Depression*Thwarted Belongingness Anxiety*Thwarted Belongingness Stress*Thwarted Belongingness				0.28
			0.33**				−0.15
			−0.18				0.56*
*R* ^2^	0.023	0.099	0.104	0.158	*R* ^2^	0.023	0.099	0.146	0.173
*R*^2^ change	0.023	0.076	0.005	0.054	*R*^2^ change	0.023	0.076	0.047	0.028
*F* change	10.01**	11.82**	2.47	8.88**	*F* change	10.01**	11.82**	23.21**	4.65**
*F*	10.01**	11.56**	9.77**	9.78**	*F*	10.01**	11.56**	14.38**	10.96**
Durbin–Watson	1.92				Durbin–Watson	1.90			

^**^ *p* < 0.01, ^*^ *p* < 0.05, + *p* < 0.10.

This study applied the Johnson–Neyman (JN) technique using CAHOST Version 1.0, which is a Microsoft Excel 2013 macro-enabled workbook, to understand the effect of the predictor variable on the dependent variable, under the influence of moderating variable (60). We refer to Carden et al. (60) for the step-by-step guide to navigating through the worksheets and report the graphics for significant interaction terms as follows. We used the workbook for significant interaction terms reported in Table 5 and were interested in the value of moderating effect where the confidence bands do not contain zero to prove the effect of X (predictor variable) and Y (outcome variable).

**Figure 2:**
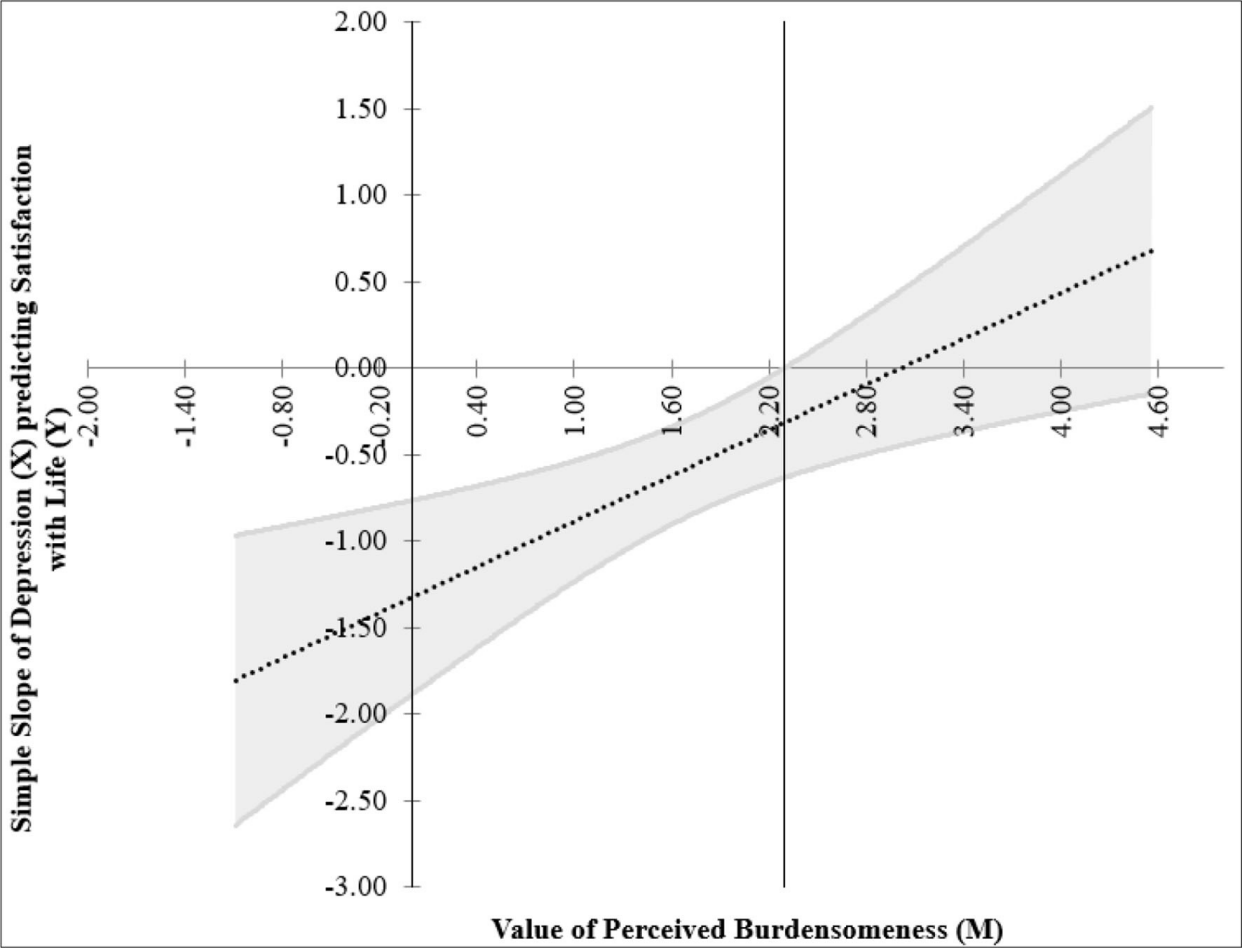
Johnson–Neyman regions representing the threshold for significant of the effects of focal predictor (depression) on the outcome variable (satisfaction with life) for different levels of moderator (perceived burdensomeness).

**Figure 3:**
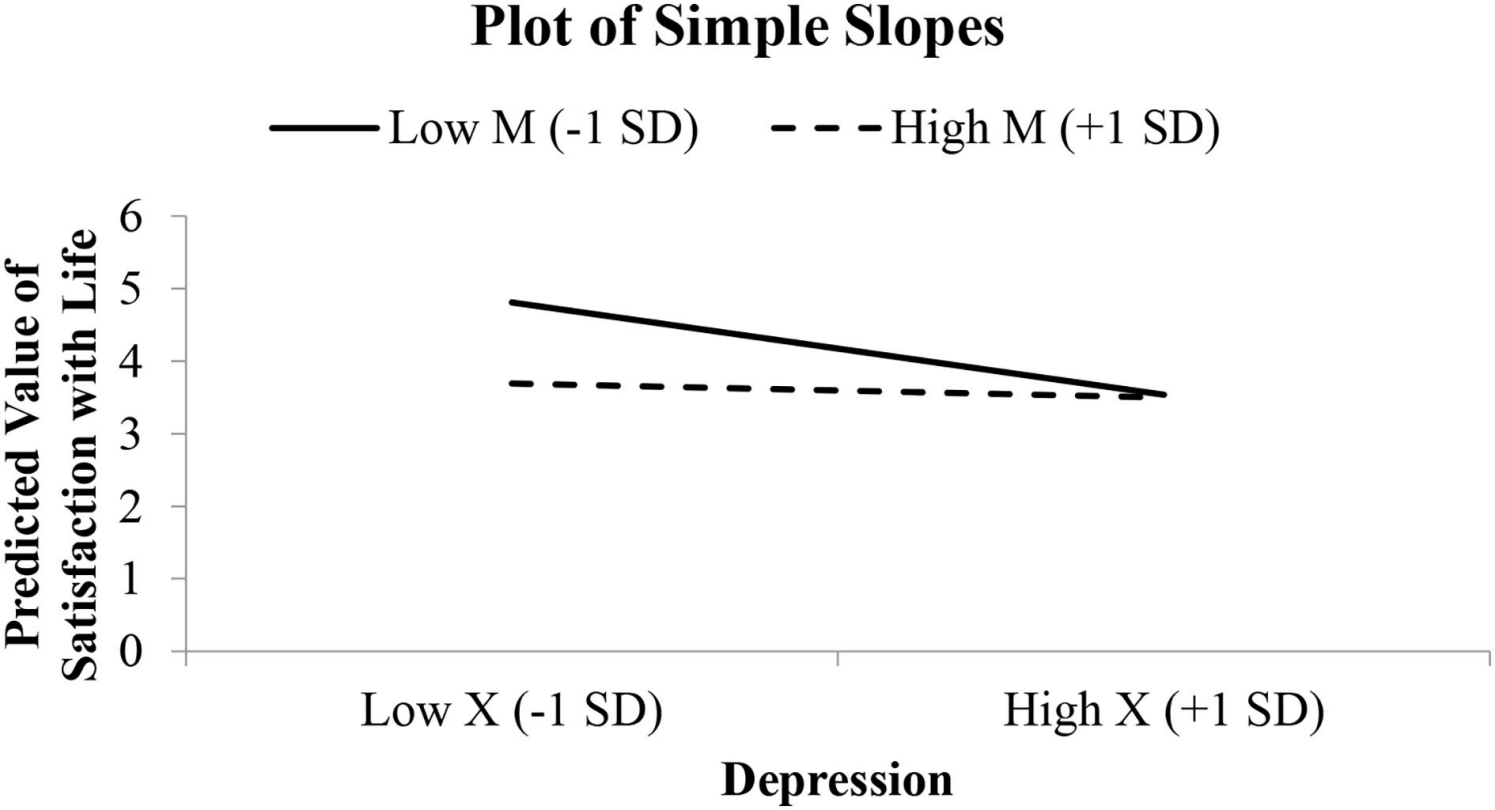
Simple slope graph for the model relating satisfaction with life to depression, perceived burdensomeness, and their interaction.

We followed the reporting method of Gorgol et al. (61), and the floodlight technique revealed that the Johnson–Neyman point (i.e., the threshold for significance of the effect of focal predictor, i.e., depression on the outcome variable, i.e., SWL) was located at 2.30 in perceived burdensomeness. This means that from low values of perceived burdensomeness up to this point, the association between depression and SWL was significant, whereas above this point, depression was not a significant predictor of SWL. The Johnson–Neyman regions presented the threshold of significance for the simple effects of depression on SWL for different levels of the moderator (perceived burdensomeness) which is shown below (Figure 2) together with the simple slope graph to show the model of interaction among satisfaction with life, depression, and perceived burdensomeness (Figure 3).

**Figure 4:**
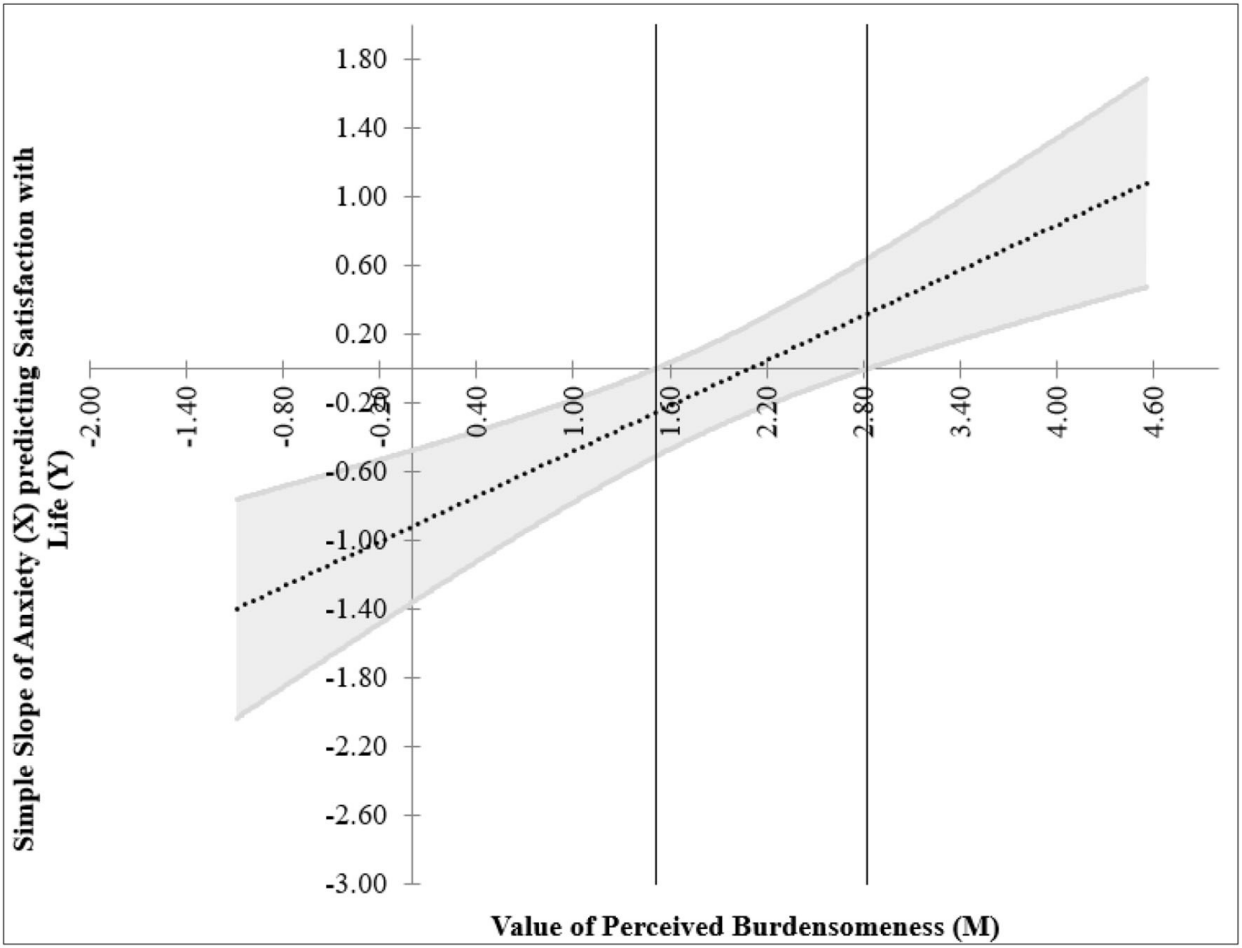
Johnson–Neyman regions representing the threshold for significant of the effects of focal predictor (anxiety) on the outcome variable (satisfaction with life) for different levels of moderator (perceived burdensomeness).

**Figure 5:**
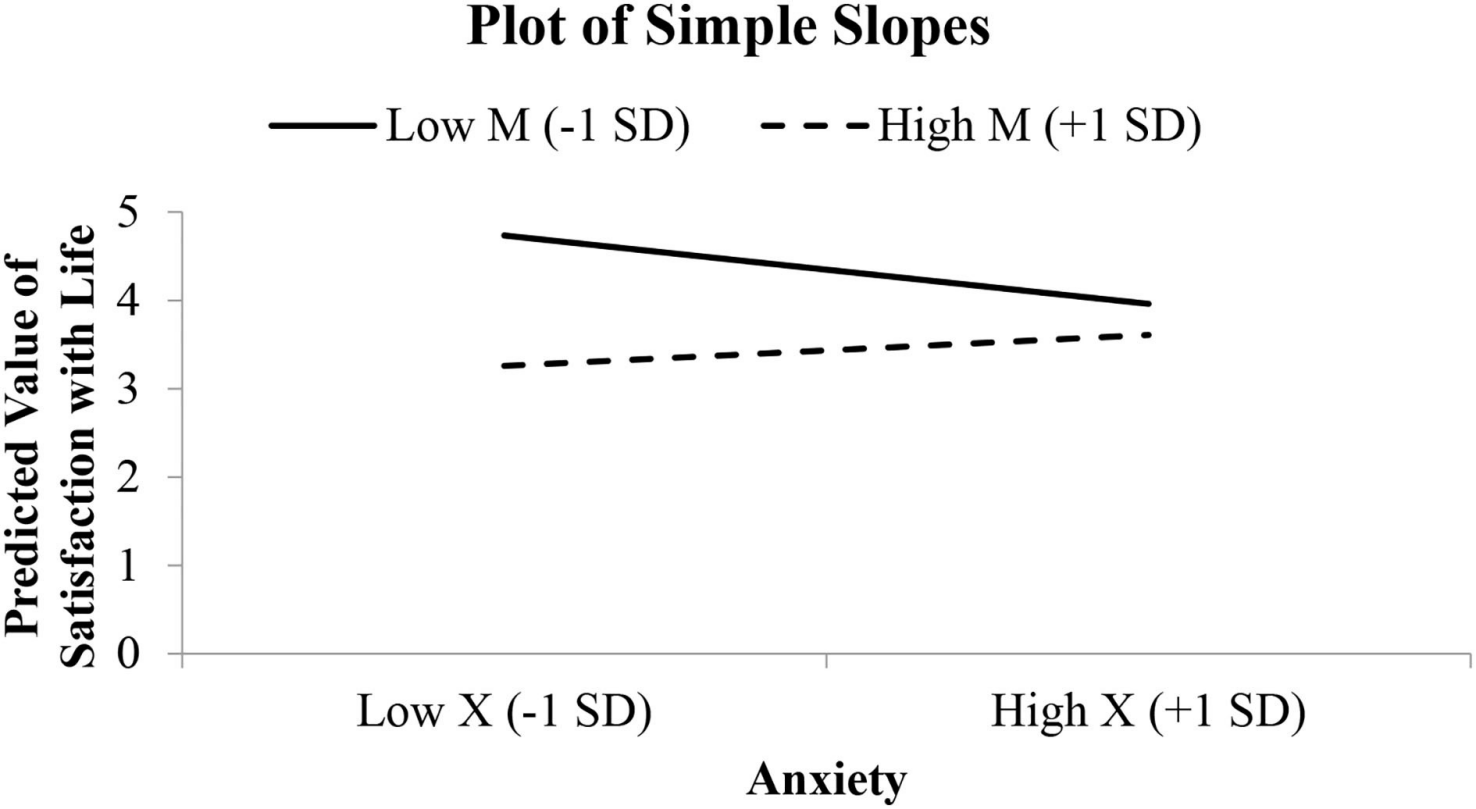
Simple slope graph for the model relating satisfaction with life to anxiety, perceived burdensomeness, and their interaction.

Figure 4 reveals that the Johnson–Neyman point (i.e., the threshold for significance of the effect of focal predictor, i.e., anxiety on the outcome variable, i.e., SWL) was located at between 1.51 and 2.82 in perceived burdensomeness. This means that the association between depression and SWL was not significant within these points, and depression was a significant predictor of SWL below and above the indicated range. The Johnson–Neyman regions presented the threshold of significance for the simple effects of anxiety on SWL for different levels of the moderator (perceived burdensomeness) which is shown below (Figure 4). The simple slope graph showed the model of interaction among satisfaction with life, anxiety, and perceived burdensomeness (Figure 5).

**Figure 6:**
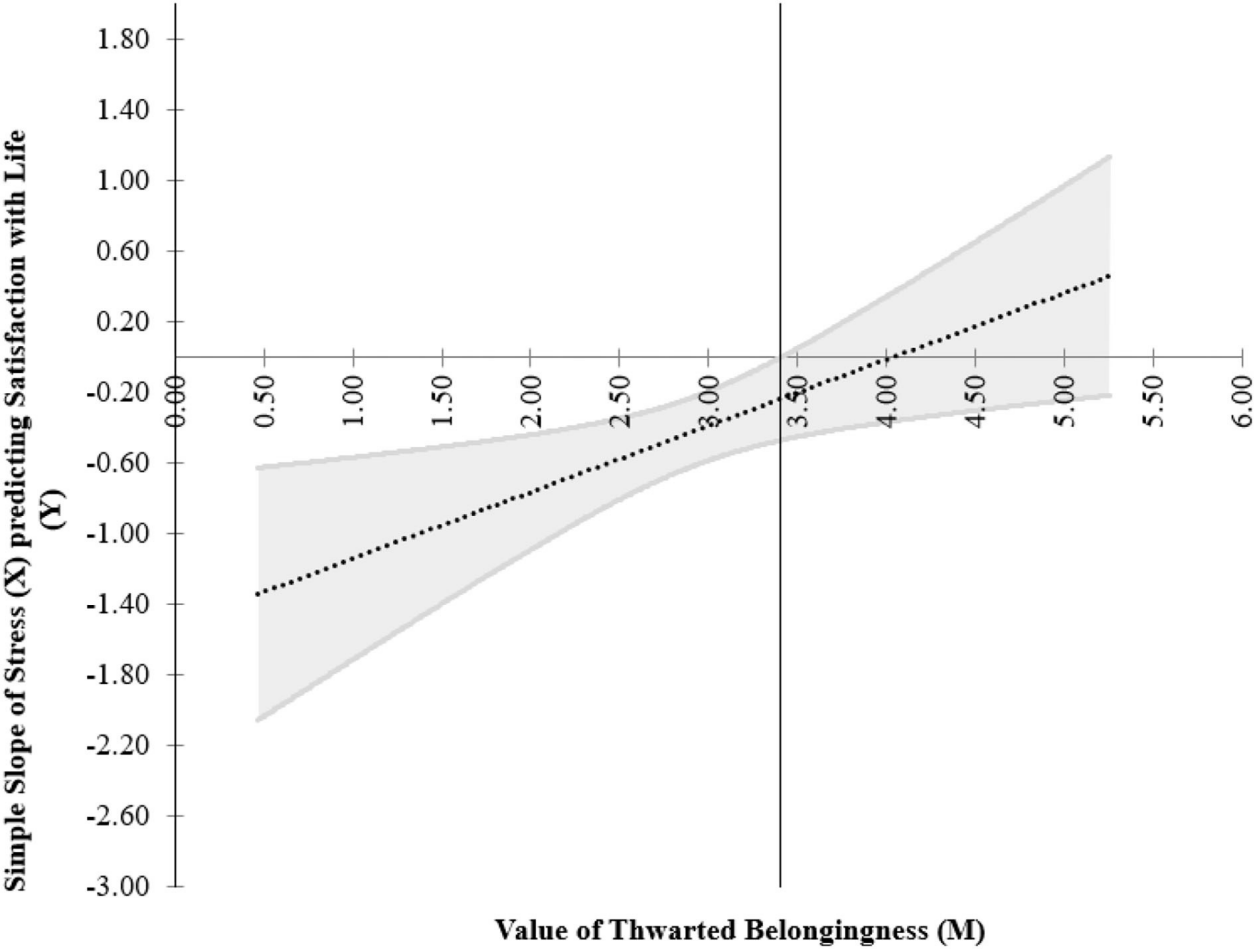
Johnson–Neyman regions representing the threshold for significant of the effects of focal predictor (stress) on the outcome variable (satisfaction with life) for different levels of moderator (thwarted belongingness).

**Figure 7:**
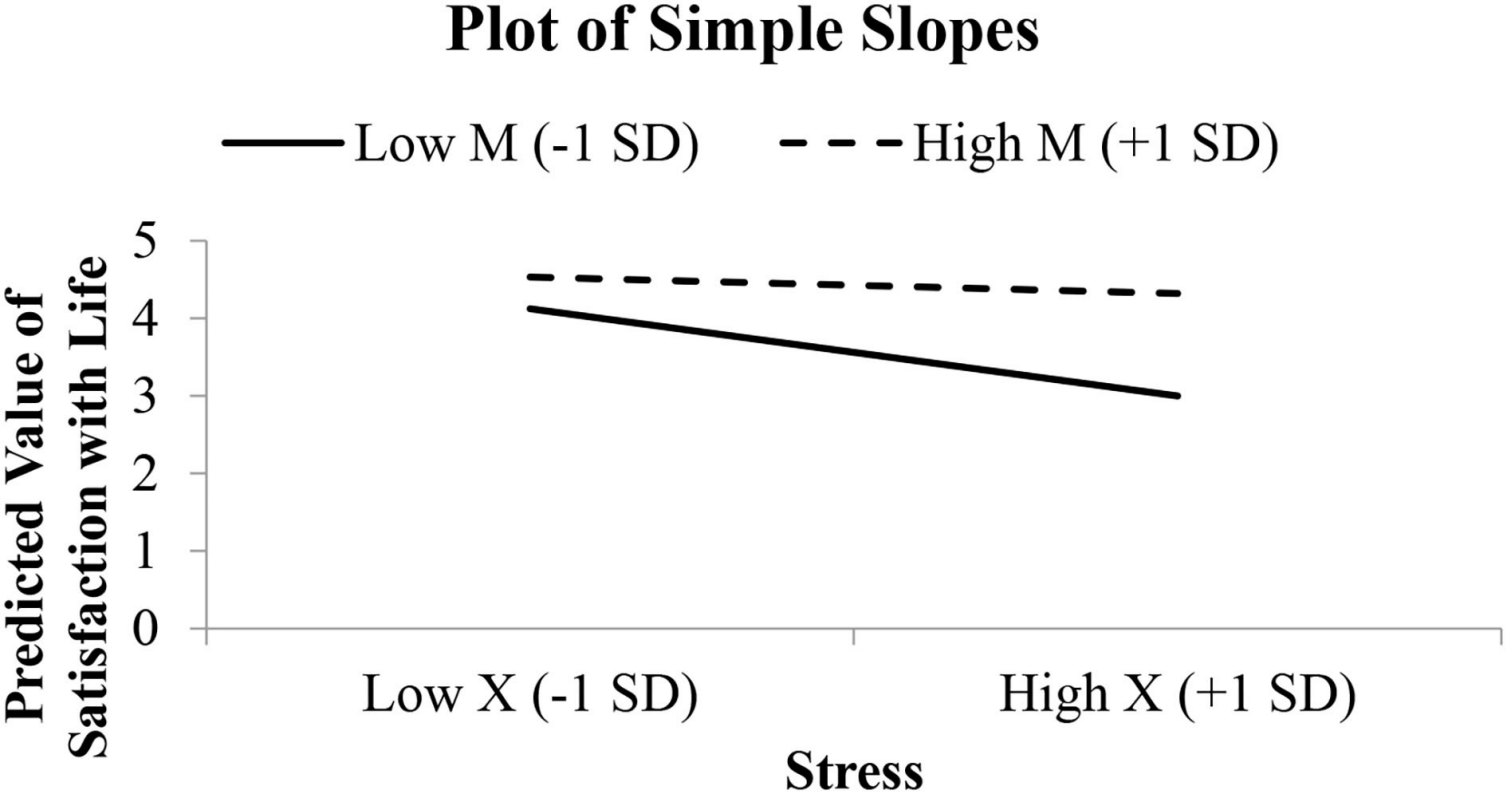
Simple slope graph for the model relating satisfaction with life to stress, thwarted belongingness, and their interaction.

The floodlight technique revealed that the Johnson–Neyman point (i.e., the threshold for significance of the effect of focal predictor, i.e., stress on the outcome variable, i.e., SWL) was located at 3.41 in thwarted belongingness. This means that from low values of thwarted belongingness up to this point, the association between stress and SWL was significant, whereas above this point, stress was not a significant predictor of SWL. The Johnson–Neyman regions presented the threshold of significance for the simple effects of stress on SWL for different levels of the moderator (thwarted belongingness) which is shown below (Figure 6). Together is the simple slope graph showing the model of interaction among satisfaction with life, stress, and thwarted belongingness (Figure 7).

## Discussion

The results of the stepwise hierarchical regression analyses presented in Table 5 show that only depression predicted SWL even though all three predictor variables, namely depression, anxiety, and stress, were negatively correlated with the SWL. The results also suggest that the moderating effect of interpersonal needs (perceived burdensomeness and thwarted belongingness) could potentially reduce or raise SWL among young adults.

Table 5 presents the results of hierarchical regression analyses, where perceived burdensomeness exhibited a full moderating effect on the relationship understudy. Perceived burdensomeness was not significant as a predictor (refer to Step 3 of Table 5) and its interaction with depression and anxiety was significant (refer to Step 4 of Table 5), thus suggesting its pivotal role in individuals' SWL.

The Johnson–Neyman approach in analyzing the interaction presents further information on where depression's effect on satisfaction with life is significant when the value of perceived burdensomeness is <2.30 (Figure 2). The interaction was not significant for individuals with the value of perceived burdensomeness above 2.30. The dotted line can be seen as nearly horizontal, agreeing with the JN graph that the effect of depression on SWL is not significant for high level of perceived burdensomeness—that is, above 2.30 (Figure 3). Figure 3 presents the simple slope to visualize the relationship between depression and SWL among two groups of individuals, where one group scored low on perceived burdensomeness and the other group scored high on perceived burdensomeness.

Figure 4 shows two regions of significance where anxiety's effect on satisfaction with life is significant when the value of perceived burdensomeness is <1.51 and >2.82. The interaction was not significant for individuals with the value of perceived burdensomeness between 1.51 and 2.82. Figure 5 presents the relationship between anxiety and SWL among two groups of individuals, where one group scored low on perceived burdensomeness and the other group scored high on perceived burdensomeness.

The negative significant relationship between (i) depression and (ii) anxiety with SWL is stronger among individuals who scored low on perceived burdensomeness. This is clearly depicted in Figures 3, 5 where individuals who reported low perceived burdensomeness were also consistently less satisfied with life. Hence, from this, we opine that a negative perception of the self as a burden to others is not beneficial for maintaining one's wellbeing and could be harmful. Our findings highlight the necessity of preventing negative mental conditions and promoting positive mental health in young adults, especially the sense of perceived burdensomeness among the individuals who reported experiencing depression or anxiety. For them, having to experience psychological distress and intensified further with a sense of perceived burdensomeness, their sense of SWL was negatively affected. Delineating the role of perceived burdensomeness as a potential risk factor to improve SWL and indirectly reduce suicidal thought is of importance for HEIs to better understand and better equip themselves to prevent any suicide risk among the students. Thus, it is important that universities and colleges can offer and encourage the agenda of mental health (62) and execute activities that focus on advocating the importance of mental wellbeing among students (63), especially programs that reduced the belief that one is a burden to others. In the United States and other countries, suicide prevention programs now focus on the theme “You Matter,” which highlights the importance and significance of developing a sense of belongingness (64).

In managing the sense of perceived burdensomeness, interventions with cognitive bias modification and psychoeducation were proven to be effective (65). Students who exhibited depression and anxiety symptoms, as well as those who perceived themselves as a burden to the family, community, or society, would require a systematic program which involved debuting the irrational belief of “I am a burden to a society.” The nature of the program should focus on managing one's self-doubt and promoting self-care management and a sense of control. For example, cognitive behavioral therapy (CBT), could be used as a form of intervention modality to restructure or reframe the irrational and self-doubt in these individuals. The use of CBT principles to correct irrational thoughts and behavior related to perceived burdensomeness helped to dispute the irrational belief of “I am a burden to my family” and “I am useless.” In the process, individuals are challenged to produce evidence which said they are of no value to society, and with the help of the programs, individuals could develop self-help strategies to dispute their negative beliefs, which are usually self-limiting beliefs in them and, thereafter, generate a new healthy self-belief in them.

Thwarted belongingness is a quasi-moderator because it was significant as a predictor (refer to Step 3 of Table 5) and its interaction with stress was significant (refer to Step 4 of Table 5 and Figure 6). The Johnson–Neyman approach in analyzing the interaction presents further information on where stress effect on satisfaction with life is significant when the value of thwarted belongingness is <3.41 (Figure 6). The interaction was not significant for individuals with the value of thwarted belongingness above 3.41. Figure 7 presents the simple slope to visualize the relationship between stress and SWL among two groups of individuals, where one group scored low on thwarted belongingness and the other group scored high on thwarted belongingness. The dotted line can be seen as nearly horizontal, agreeing with the JN graph that the effect of stress on SWL is not significant for high level of thwarted belongingness (Figure 7). This negative significant relationship is stronger among individuals who scored low on thwarted belongingness. Our study revealed that in the presence of stress, regardless of levels, individuals with low thwarted belongingness experienced a lower level of SWL as compared to those with a high level of thwarted belongingness. The thwarted belongingness is a negative thing that needs to be addressed by encouraging social attachment, especially among students who scored high on stress.

With the advent of the digital age, young adults who are becoming less socially attached put their SWL at risk in the presence of higher stress. Young adults today are experiencing lesser social interaction as they prefer virtual interaction (66), with a desire to escape from physical realities (67, 68). This could be attributed to young adults' desire for independence while exhibiting an inclination to withdraw and isolate themselves from others, gradually reducing their social circles, and limiting their ability to seek social support when needed (69). In addition, they enveloped themselves with an escapism behavior by spending long hours within the virtual world, through imagined ideal virtual relationships. This trend affects young adults' thwarted belongingness. Stress is inevitable, and in the presence of thwarted belongingness, SWL appeared to be higher. This study recommends the importance of managing level of thwarted belongingness to manage the effect of stress on SWL.

Strengthening the sense of community and belongingness to increase the SWL among the individuals is vital. University students, who are more involved and feel belong, cope better with stress and have a better overall mental health status (24). A sense of belonging in an educational environment begins with peers and extends to the classroom, or beyond the campus. The sentiment is critical for students learning and development and contributes to positive student experiences. To instill, a higher level of thwarted belongingness among the university students in the learning and teaching context of COVID-19 and post-pandemic would require additional preparedness, design, and adequate resource allocation. The HEIs need to emphasize the importance of an inclusive climate and promote greater understanding and acceptance of diversity and differences. Furthermore, universities are comprised of members from diverse backgrounds and cultures, and each culture could be unique to each individual. The university community must be aware of the need to create a safe and engaging space in which these students can participate and no one felt being left behind.

Our finding, however, contrasts Çivitci (70)s study, which found that undergraduate students who participate more in extracurricular activities have higher belongingness and higher life satisfaction. Similarly, a study by Mellor (71) supported the idea of the “belongingness hypothesis,” suggesting that individuals tend to form long-term, meaningful, and positive relationships while failure to achieve this can lead to social isolation, loneliness, and suicidal thoughts. These inconsistencies show that it is essential to be cautious in concluding the relationship between the need for belongingness and SWL.

## Limitations and suggestions for future study

The study is not without limitations. The DASS instrument is not equivalent to clinical diagnosis although DASS has been used and validated in various settings. Although our study reported the positive association between satisfaction in life and thwarted belongingness, we recommend our findings be interpreted with caution and limit the generalizability of the results. Replications of this study in various settings may be necessary.

Our participants are students who the enumerator has accessed, and they were approached in their respective education settings; thus, this poses a risk of social desirability bias and influences their responses to the questionnaire. Future studies may formulate online surveys which allowed better anonymity. In addition, this is a cross-sectional study which does not allow for causal–effect relationships to be determined or generalized. Thus, future studies should use broader sampling and include evidence obtained from objective data, such as the students' records and data (e.g., Facebook and Instagram posts which may project their state of wellbeing).

Past randomized controlled trial (RCT) program by Van Orden (72) where seniors were paired with peer companions was shown to significantly increase the sense of social connectedness among participants. Given the success of this program and as compared to the research design of this present study (i.e., cross-sectional), researchers or educators could replicate the study where pairing exercises could be used as a method to promote a greater sense of inclusiveness in an RCT setting. RCT protocol allowed for assessments to be measured systematically over a period of time and educators would be able to trace changes in scores over time and allow immediate intervention for university students or young adults if needed as compared to cross-sectional research design which failed to evaluate the effectiveness of intervention over time.

Interestingly, we found a positive relationship between thwarted belongingness and SWL, which is in contrast to findings from past research. We attribute this unique finding to the fact that our participants are mostly “Generation Z” digital natives; hence, their lives are vastly authored by digital technology influence. These include their social and communication (i.e., through online platforms), and their sense of belongingness may be developed quite differently compared to other generations. At the point of writing, generational variance has not been given enough attention in clinical and teaching settings. We believe the concept of generational differences should be examined further in research and that mental health practitioners, as well as teaching staff, should be more aware of its effects.

In addition, future studies may also include variables such as social online behavior (e.g., types, duration, and who the users communicate virtually with) as variables and examine the relationship of these variables on SWL. Future studies may extend the current study's framework and could consider incorporating measurement of suicidal risk and behavior as the sequel to SWL, as evidenced by past studies.

## Conclusion

Our study aimed to examine the relationship between depression, anxiety, stress, and SWL among university students in Malaysia. Furthermore, we explored the moderating effect of interpersonal needs, specifically perceived burdensomeness and thwarted belongingness on this relationship. Perceived burdensomeness is indeed a significant moderator of the relationship between (i) depression, and (ii) anxiety and SWL. On the contrary, thwarted belongingness exerts a significant moderating effect on the relationship between stress and SWL. Our research sets the stage for future researchers to investigate mental health conditions further, with a focus on the role of interpersonal needs.

## Data availability statement

The raw data supporting the conclusions of this article will be made available by the authors, without undue reservation.

## Ethics statement

The studies involving human participants were reviewed and approved by IRB 2018/044. The patients/participants provided their written informed consent to participate in this study.

## Author contributions

KS: conceptualization and data analysis. KS, CC, and PB: methodology. CC and KS: data collection. KS, CC, DO, and PB: drafting and final writing. All authors contributed to the article and approved the submitted version.

## Funding

The authors received financial support for the publication of this article from Sunway University.

## Conflict of interest

The authors declare that the research was conducted in the absence of any commercial or financial relationships that could be construed as a potential conflict of interest.

## Publisher's note

All claims expressed in this article are solely those of the authors and do not necessarily represent those of their affiliated organizations, or those of the publisher, the editors and the reviewers. Any product that may be evaluated in this article, or claim that may be made by its manufacturer, is not guaranteed or endorsed by the publisher.
